# Parallel Catalyst Synthesis Protocol for Accelerating Heterogeneous Olefin Polymerization Research

**DOI:** 10.3390/polym15244729

**Published:** 2023-12-17

**Authors:** Patchanee Chammingkwan, Mostafa Khoshsefat, Minoru Terano, Toshiaki Taniike

**Affiliations:** Graduate School of Advanced Science and Technology, Japan Advanced Institute of Science and Technology, 1-1 Asahidai, Nomi 923-1292, Japan; mostafa@jaist.ac.jp (M.K.); terano@jaist.ac.jp (M.T.);

**Keywords:** Ziegler-Natta catalyst, parallel synthesis, miniature, morphology, olefin polymerization

## Abstract

The data scientific approach has become an indispensable tool for capturing structure–performance relationships in complex systems, where the quantity and quality of data play a crucial role. In heterogeneous olefin polymerization research, the exhaustive and multi-step nature of Ziegler-Natta catalyst synthesis has long posed a bottleneck in synthetic throughput and data generation. In this contribution, a custom-designed 12-parallel reactor system and a catalyst synthesis protocol were developed to achieve the parallel synthesis of a magnesium ethoxide-based Ziegler-Natta catalyst. The established system, featuring a miniature reaction vessel with magnetically suspended stirring, allows for over a tenfold reduction in synthetic scale while ensuring the consistency and reliability of the synthesis. We demonstrate that the established protocol is highly efficient for the generation of a catalyst library with diverse compositions and physical features, holding promise as a foundation for the data-driven establishment of the structure–performance relationship in heterogeneous olefin polymerization catalysis.

## 1. Introduction

Heterogeneous Ziegler-Natta catalysts play a crucial role in the industrial production of polyolefins. They function at two distinct scales: the active site level, where the formation and nature of Ti active species on MgCl_2_ surfaces govern polymerization activity and polymer microstructure [1,2,3,4,5,6], and the particle scale, where the pore structure and morphology impact polymerization kinetics and the morphology of polymer particles through phenomena such as fragmentation and replication [7,8,9]. Despite their long-standing commercial success, the multi-component nature of hierarchical particle architectures, coupled with dynamic changes spanning over the polymerization time scale, gives rise to a complex interplay in the polymerization process. This complexity poses a challenge in establishing a clear correlation between catalyst structure and performance, making trial and error the primary approach in catalyst development.

In recent years, the data scientific approach has become an essential tool in capturing structure–performance relationships in a complex system [10,11,12,13,14,15]. This approach proves invaluable in unraveling the intricate interplay, particularly when systematic control over individual factors is challenging. However, the significance of a dataset cannot be overstated, as a substantial number of robust data are pivotal for establishing reliable correlations [14,15]. Likewise, the high-throughput experimentation concept has emerged as a powerful tool for rapid data generation. In olefin polymerization research, high-throughput experimentation has been addressed across several stages of experimentation, including creation of homogeneous catalyst libraries and, in some cases, their heterogenized counterparts through combinatorial ligand and metal-ligand variations [16,17,18,19,20,21,22]; polymerization performance evaluation using microscale arrays of reaction vessels [16,17]; pooled polymerization [18,19]; high-precision parallel pressure reactor modules [23,24,25,26,27,28,29,30,31]; and characterization processes for rapid access to polymer properties [25,26], especially from a small polymer quantity. The implementation of this concept has led to the successful uncovering of new homogeneous catalyst structures [16,17,20,32] and has facilitated the exploration of external donors used in heterogeneous Ziegler-Natta catalyst systems [27]. Nevertheless, challenges arise when extending this methodology to the synthesis of Ziegler-Natta solid catalysts. A careful balance of precision and instrumental design is required due to the complicated nature of the synthesis involving many steps; extensive use of corrosive and hygroscopic chemicals; rigorous control over inert atmosphere; and control over macroparticle morphology, etc. Currently, an exhaustive and time-consuming one-by-one approach, typically requiring a continuous 12 h per batch, is practiced in both academia and industry, serving as a bottleneck in heterogeneous olefin polymerization research.

Our study addresses this gap by achieving controlled parallel synthesis of solid catalysts. We introduce a custom-designed 12-parallel reactor system and a catalyst synthesis protocol that enable the synthesis of a magnesium ethoxide-based Ziegler-Natta catalyst with enhanced throughput, at an affordable cost even for an academic laboratory. In this report, we present the method and protocol for each developmental stage step-by-step and address key considerations towards achieving synthetic control. Finally, we demonstrate the utilization of the developed protocol for generating a Ziegler-Natta catalyst library, paving the way for exploring the structure–performance relationship in olefin polymerization catalysis through statistical means.

## 2. Materials and Methods

### 2.1. Materials

Magnesium powder (Mg, particle size = 0.06–0.3 mm) was purchased from Merck KGaA, Darmstadt, Germany. Iodine (I_2_, purity > 99.0%, Merck KGaA) was used as an initiator. Ethanol (purity > 99.5%), *n*-heptane (purity > 99.0%) and toluene (purity > 99.0%) were purchased from Tokyo Chemical Industry Co., Ltd., Tokyo, Japan. They were dried over a 3A molecular sieve, followed by N_2_ bubbling. Titanium tetrachloride (TiCl_4_, special grade) and di-*n*-butyl phthalate (DBP, purity > 98.0%) were purchased from Wako Pure Chemical Industry Ltd., Richmond, VA, USA and used as received. Polymerization-grade propylene was donated by Japan Polypropylene Co., Ltd., Tokyo, Japan. Triethyl aluminum (TEA) as a cocatalyst was donated by Tosoh Finechem Corp., Shunan, Japan and used as a diluted solution in heptane. Cyclohexyl (dimethoxy) methylsilane (CMDMS, purity > 98.0%) as an external donor was purchased from Tokyo Chemical Industry Co., Ltd. and used as a diluted solution in heptane.

### 2.2. Synthesis of Magnesium Ethoxide as a Catalyst Precursor

**Figure 1:**
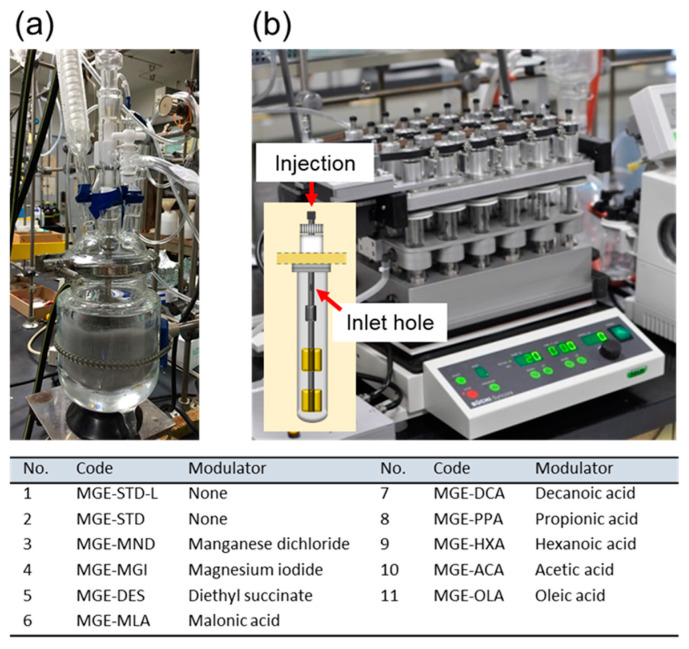
Reactor setups for the synthesis of magnesium ethoxide as a catalyst solid precursor: (**a**) large-scale synthesis, and (**b**) small-scale parallel synthesis (adapted with permission from ref. [35] Copyright 2017 American Chemical Society). Inserted table lists the codes of MGE used for catalyst preparation. Note that all samples, except for MGE-STD-L, were synthesized using the parallel reactor setup in (**b**).

Spheroidal magnesium ethoxide (MGE) was synthesized as a solid precursor for a Ziegler-Natta catalyst. Two setups were employed for the preparation of MGE with different quantities: large-scale synthesis (~100 g per batch) using a jacket-type 500 mL glass reactor equipped with a condenser and an overhead motor stirrer (Figure 1a) [33,34], and small-scale synthesis (~4 g per batch) using a 24-parallel reactor system established in our previous work (Figure 1b) [35]. In a typical large-scale synthesis, 0.68 g of I_2_ and 35 mL of ethanol were charged into the reactor under N_2_ atmosphere. The temperature was raised to the reflux temperature under stirring at 180 rpm. After the dissolution of I_2_, 3.0 g of Mg powder and 35 mL of ethanol were repetitively added nine times with a time interval of 10 min. The reaction was allowed to continue for 2 h at the reflux temperature under N_2_ flow to eject H_2_ generated from the reaction. The resultant product was washed twice with heptane, transferred to a round-bottom flask, and dried under vacuum (denoted as MGE-STD-L). The small-scale synthesis adopted a condition and procedure slightly different from those of the large-scale synthesis. At first, a repetitive cycle of evacuation and N_2_ purging was performed to prepare the N_2_ atmosphere. When the temperature reached 75 °C, 3.0 mL of an I_2_ solution in ethanol (0.13 mol L^−1^) was introduced to each reaction vessel under N_2_ flow. After stirring at 250 rpm for 10 min, 0.25 g of Mg powder suspended in 3.0 mL of ethanol was added 5 times via a plastic syringe with a time interval of 30 min. To produce MGE with varied particle characteristics while using the same synthesis condition, we employed a modulator approach. This involved adding 0.1 mmol of a modulator in 1.0 mL of ethanol immediately after each precursor addition (5 times in total, equivalent to 1 mol% of a modulator relative to Mg). After the last addition, the reaction was continued for 3 h, and then the solid was washed twice with 20 mL of heptane. The reaction vessels were removed from the reactor module and the solid content was dried in parallel using a centrifugal vacuum evaporator (CVE-3100, EYELA). The codes of the synthesized MGE are provided in the inserted table in Figure 1 along with the type of modulators used.

### 2.3. Synthesis of Catalyst

The catalyst synthesis procedure involved the following steps [33,34] in a typical laboratory setting. In a 300 mL three-neck round bottle flask equipped with a condenser and an overhead motor stirrer, 15 g of MGE as a solid precursor and 150 mL of toluene were introduced under N_2_ atmosphere. After cooling to 5 °C using an ice bath, 30 mL of TiCl_4_ was added dropwise via a dropping funnel over a period of 1 h. The suspension was then slowly heated to 90 °C using an oil bath. Following the addition of 4.5 mL of DBP, the temperature was raised to 110 °C, and the reaction was maintained for 2 h. The solid was washed twice with toluene via decantation. The second treatment was conducted by adding 30 mL of TiCl_4_ in 150 mL of toluene. The reaction was carried out at 110 °C for another 2 h. The resulting product was repetitively washed with toluene followed by heptane and finally dried under vacuum at room temperature. The protocol for parallel catalyst synthesis essentially followed the laboratory-scale mentioned above with some optimizations. Further details will follow below. 

### 2.4. Characterization

The morphology of catalyst particles was observed on a scanning electron microscope (SEM, Hitachi S-4100, Hitachi High-Tech Corporation, Tokyo, Japan) operated at an accelerate voltage of 20 kV. A sample was dispersed on carbon tape in a glove bag under N_2_ atmosphere. Particle characteristics were acquired by analyzing SEM images using ImageJ software. The titanium (Ti) content of a catalyst was determined based on a colorimetric method using ultraviolet-visible spectrometry (UV-vis, JASCO V670, JASCO Corporation, Tokyo, Japan). A catalyst (50 mg) was dissolved in a solution of HCl and H_2_SO_4_. Thereafter, 200 µL of H_2_O_2_ (35% aqueous solution) was added to form a titanium peroxocomplex. The absorbance intensity at 410 nm was then employed to determine the Ti content based on a calibration curve acquired using a Ti standard solution. The organic content was analyzed based on ^1^H NMR (Bruker AVANCE III 400 MHz, Bruker Corporation, Billerica, MA, USA) in a solution state using dimethyl sulfoxide-d_6_ as a solvent and an internal lock, and 1,1,2,2-tetrachloroethane as an internal standard. N_2_ adsorption/desorption experiments were performed on a BELSORP Max (BEL Japan, Tokyo, Japan) instrument at 77 K. An amount of 20–30 mg of catalyst was loaded into a glass cell in an N_2_ glove bag. Degassing was performed under vacuum at 80 °C for 2 h prior to the measurement. The pore size distribution was acquired from the adsorption branch based on the NL-DFT method.

### 2.5. Polymerization Performance Evaluation

Propylene polymerization was conducted in a slurry phase using heptane as a solvent. In a 1 L stainless steel high-pressure reactor equipped with a mechanical stirrer, 300 mL of heptane, 3.0 mmol of TEA as a cocatalyst and impurity scavenger, and 0.30 mmol of CMDMS as an external donor were added under N_2_ atmosphere. The solvent was then saturated with propylene at 50 °C and 0.4 MPa for 30 min. Thereafter, a measured amount of catalyst was injected into the reactor to initiate polymerization. The polymerization proceeded for 1 h at 50 °C and 0.4 MPa before being terminated by depressurization. The resultant polymer was collected, washed with ethanol, and dried in a vacuum at 60 °C for 6 h. The isotacticity of the polymer was evaluated based on its xylene-insoluble content (XI). 1.0 g of the polymer was dissolved in 50 mL of xylene containing 0.03 wt% of butylated hydroxytoluene as a stabilizer. The temperature was raised to 135 °C and held for 1 h for complete dissolution. Thereafter, the solution was slowly cooled down to 20 °C over a period of 2 h to precipitate the insoluble part. An amount of 20 mL of the supernatant was collected and dried under vacuum at 60 °C for 6 h. The insoluble fraction was calculated based on Equation 1:

XI (wt%) = (1 − 5a/2) × 100(1)

where a is the weight of polymer obtained after drying the supernatant.

## 3. Results and Discussion

### 3.1. Setup and Protocol for Catalyst Synthesis

#### 3.1.1. Downsizing

**Figure 2:**
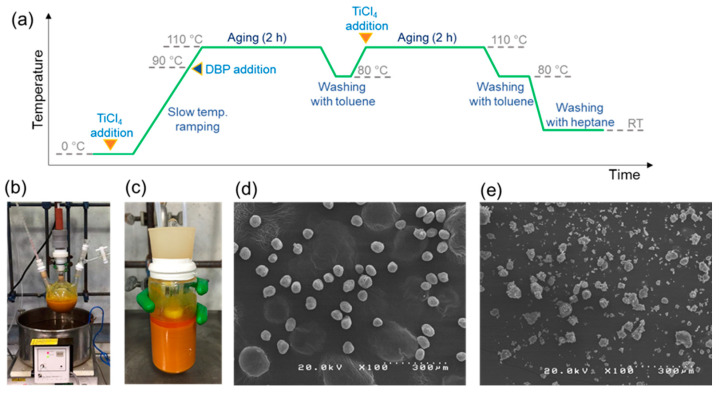
Catalyst synthesis setup and procedure: (**a**) typical procedure for catalyst synthesis; (**b**) conventional laboratory reactor setup; (**c**) 30 mL miniature reaction vessel equipped with a suspended magnetic stir bar; (**d**) SEM image of a catalyst synthesized using the miniature reaction vessel; and (**e**) SEM image of a catalyst synthesized using the same miniature reaction vessel but with an unsuspended magnetic bar (leading to morphological failure).

The first step in developing our parallel catalyst synthesis protocol was reducing the size of the reaction vessel, targeting a synthesis quantity of 1 g per batch, which is the minimum required for full package characterization and performance evaluation. A typical step for conventional catalyst synthesis is shown in Figure 2a. The reaction advances through the solid-state conversion of MGE into MgCl_2_, using an excessive quantity of TiCl_4_ as the chlorinating agent. As chlorination occurs, TiCl_4_ is concurrently adsorbed onto the undercoordination surfaces of formed MgCl_2_ nanocrystals, serving as active sites. A Lewis base (DBP in this case) is added as an internal donor to direct the growth of nanocrystals and surface exposures, as well as to modify the stereo and electronic properties of Ti species through coadsorption. Reaction by-products such as Ti alkoxy and phthaloyl chloride, which are generated from the reaction between TiCl_4_ and MGE, and between TiCl_4_ and DBP, are removed by repetitive washing. Unlike a homogeneous catalyst system, the first crucial determinant of the process’s success for the heterogeneous catalyst system lies in controlling the macroparticle morphology. The morphology of the catalyst particles significantly impacts the entire polyolefin production process. It exerts influence over the kinetics of polymerization and the overall catalyst performance through the fragmentation process. Additionally, replication phenomena between catalyst and polymer particles makes the initial catalyst morphology important in determining the final polymer morphology. A commendable approach to maintaining the integrity of catalyst macroparticles involves the implementation of a non-destructive stirring technique and a temperature control strategy. In the laboratory synthesis of an MGE-based catalyst utilizing conventional glassware (as depicted in Figure 2b), a mechanical stirrer is utilized to agitate the suspension without crushing the particle. TiCl_4_ is added slowly and carefully under N_2_ atmosphere at a low temperature to control the heat produced by the exothermic reaction. Afterward, the temperature is slowly increased to the desired temperature to complete the reaction while reducing the risk of particle breakage. Downsizing the reaction vessel from that of conventional glassware necessitates compliance to these basic strategies. Nevertheless, the confined space makes traditional mechanical stirring impractical. Here, we utilized a magnetic suspended stirring system, which suits the dimensions of a compact reaction vessel (Figure 2c). Dropwise addition of TiCl_4_ with a controlled addition rate was enabled via a peristaltic pump, where the temperature was controlled using an oil bath. Leveraging the miniature reaction vessel and suspended magnetic stirring system enabled a great reduction in the synthetic scale, i.e., from a standard 15 g per batch in a 300 mL round-bottom flask to 1 g per batch in a 30 mL reaction vessel, which significantly reduced the amount of chemicals used and waste produced. The successful catalyst synthesis was demonstrated by the excellent particle morphology (Figure 2d), comparable to results achieved through conventional glassware [33,34]. Notably, this level of morphological control was never attainable with a typical magnetic stirring system, as shown in Figure 2e.

#### 3.1.2. Parallelization

**Figure 3:**
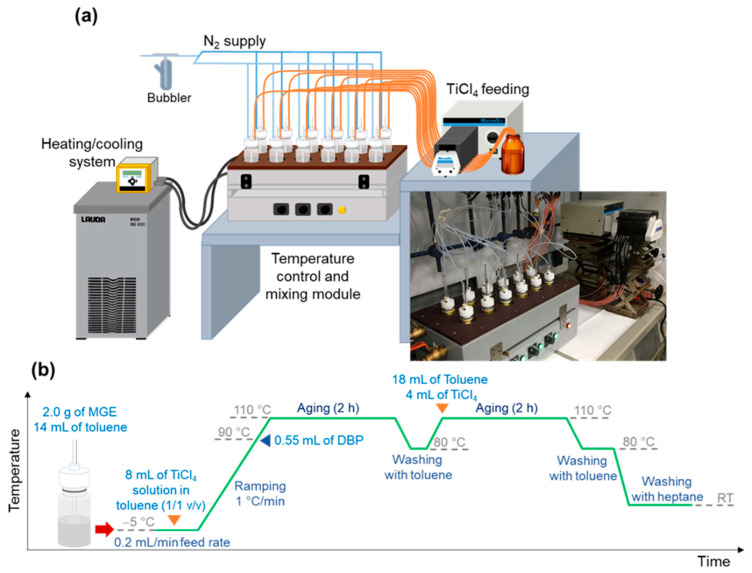
Parallel catalyst synthesis protocol: (**a**) illustration of the entire reactor system and the actual image of the module, and (**b**) optimized condition for the parallel synthesis of 12 catalysts using a 50 mL reaction vessel.

In the next step, we developed a system for controlling the temperature and mixing for the synthesis of twelve catalysts in parallel using the above-developed miniature reaction vessels (Figure 3). The system involved a closed oil circulation bath, featuring twelve holes for accommodating the reaction vessels in the upper section and a belt-drive magnetic stirring system in the lower section. Temperature control was achieved by circulating oil from a separate heating/cooling unit (Eco RE630, LAUDA), allowing precise regulation of the heating rate and temperature within the range of −20 °C to 150 °C. Figure 3a illustrates the entire parallel reactor setup, where a miniature reaction vessel was enhanced with a custom-designed Teflon cap integrated with a magnetic suspended stir bar. The Teflon cap also featured two holes at the top for reactant feeding and N_2_ atmosphere control. Note that the volume of the reaction vessel was interchangeable, with the vessel size of 30 mL and 50 mL for the minimum synthesis scales of 1 g and 2 g per batch, respectively. To facilitate the automatic parallel feeding of TiCl_4_, a cartridge pump (Masterflex L/S, Cole Parmer) was employed for supplying the TiCl_4_ solution to the corresponding reaction vessels. The optimized synthesis condition for the parallel catalyst synthesis protocol using a 50 mL reaction vessel is illustrated in Figure 3b.

**Figure 4:**
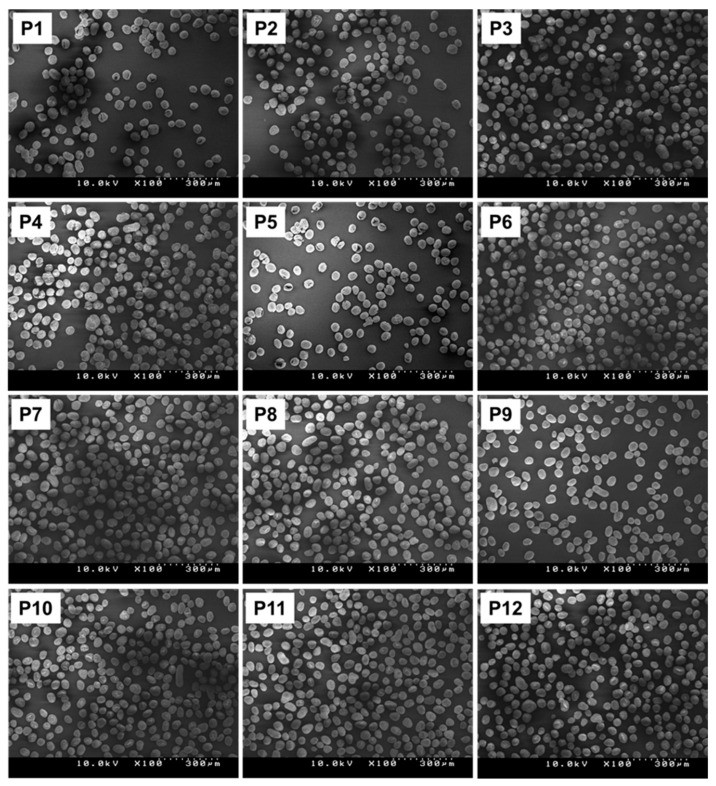
SEM images of catalysts prepared in parallel under identical conditions.

The replication of the catalyst synthesis from each position of the module was verified through the parallel synthesis of twelve catalysts under an identical condition using MGE-STD-L as a solid precursor (labeled as P1–P12). SEM images in Figure 4 show that all the synthesized catalysts possessed excellent spheroidal morphology and integrity of the particles. The absence of fines indicated effective control over the agitation and heat released during the reaction. Quantitative analyses on the particle characteristics and chemical compositions of catalysts revealed that all the properties were closely clustered around the mean value, ensuring consistency and reliability of the established setup and protocol (Table 1).

### 3.2. Generation of the Catalyst Library

**Figure 5:**
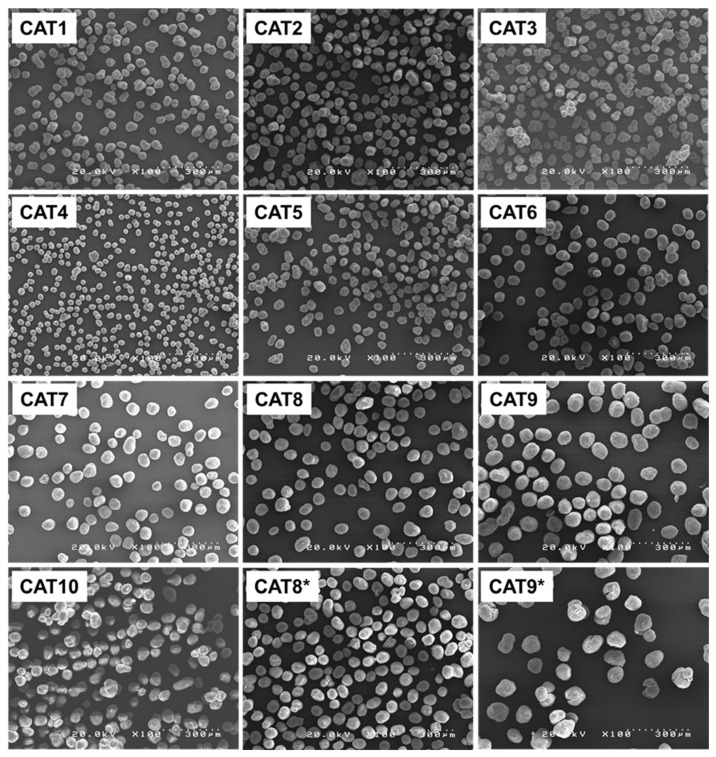
SEM images of catalyst samples prepared using different sources of MGE samples as a solid precursor. The * symbol indicates the reproduction test.

In pursuing a complicated structure–performance relationship in heterogeneous catalysis, the initial and crucial task involves creating a comprehensive catalyst library. This step has long posed a bottleneck in heterogeneous olefin polymerization catalysis primarily due to its exhaustive nature and the substantial time demands of the synthesis step. Parallel synthesis offers a remedy and can easily work on producing a catalyst library with an assortment of diverse catalyst compositions through various means, including alteration of reagent ratios, and incorporation of different types of internal donors or different reagents etc. Furthermore, diverse physical features such as particle sizes and pore characteristics can also be achieved through the alteration of the solid precursor. In our previous work, a 24-parallel reactor system was established and adopted for the synthesis of MGE as a solid precursor (see Figure 1b), in which a modulator approach was introduced to create MGE with difference particle sizes and morphologies [35]. We have reported preliminary results for the elucidation of the structure–performance relationship in propylene polymerization from six catalysts. In combination with the parallel catalyst synthesis protocol established in this work, a catalyst library can be effectively expanded. Here, 12 catalysts were prepared from different MGE sources. The first 10 catalysts (CAT1–CAT10) were prepared using 10 different MGE samples which were synthesized in parallel. Given the large variation in the particle size of the MGE sources, ranging from 33 µm to 80 µm, reproduction was also tested for CAT8 and CAT9, which employed MGE with a relatively large particle size (named as CAT8*and CAT9*). Figure 5 depicts SEM images of the resultant catalysts prepared using the developed system. Good integrity of catalyst particles was observed for all the samples despite variations in the MGE sources. The particle characteristics were basically similar to those of the corresponding MGE samples, except for the fact that catalyst particles were in general rounder and smoother due to partial dissolution of MgCl_2_ in the presence of TiCl_4_ and the internal donor.

**Figure 6:**
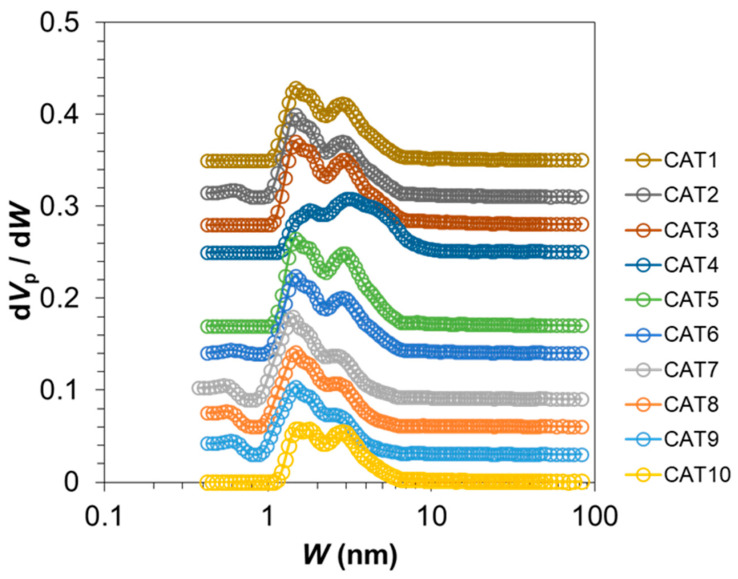
Pore size distribution of synthesized catalysts analyzed based on the NL-DFT method. *V*_p_ and *W* stand for pore volume and pore width, respectively.

Table 2 summarizes the particle characteristics and chemical composition of the catalysts. It can be observed that the different types of solid precursors led to variations not only in the physical features, but also in the chemical composition. Furthermore, the two samples demonstrated successful reproduction, implying the potential applicability of the established setup and protocol to different types of solid support. Micropores and mesopores of the catalysts were analyzed via N_2_ adsorption/desorption experiments. The distribution of pore sizes for CAT1–CAT10, as well as the pore volume data, are visualized in Figure 6 and enumerated in Table 3. Evidently, most of the catalyst porosity arose from mesopores, and distinct distributions (e.g., for CAT4 and CAT10) were observed, further rationalizing our goal in establishing a catalyst library with diverse features.

**Figure 7:**
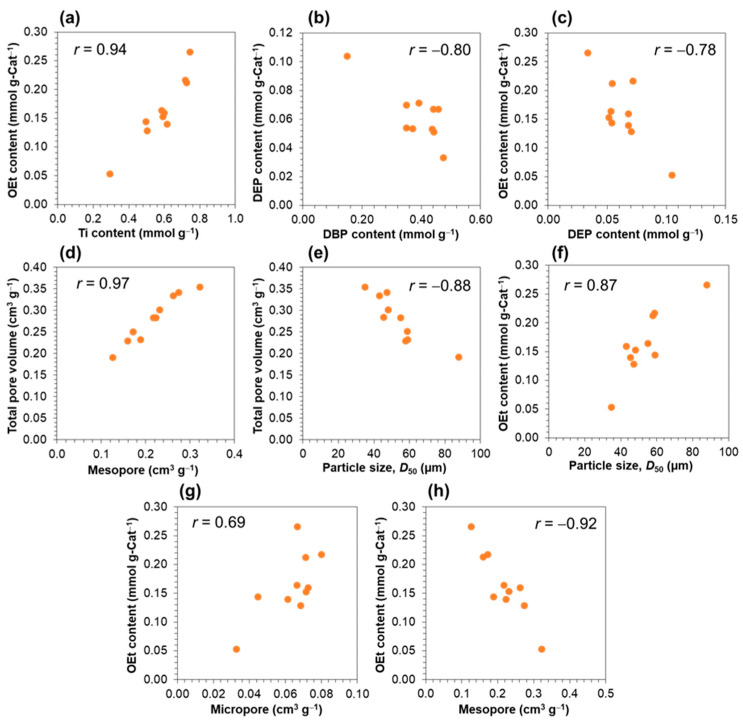
Relationships between catalyst features: (**a**–**c**) chemical compositions, (**d**,**e**) physical features, and (**f**–**h**) interrelationships between chemical compositions and physical features. The correlation coefficients (r) are integrated into their respective plots.

In an attempt to investigate the relationships among catalyst features, we conducted a correlation coefficient analysis and presented the resultant plots in Figure 7. Among the chemical composition features, a high Ti content was found to be strongly related to a high OEt content (Figure 7a), while the DEB content exhibited inverse correlations with the DBP content (Figure 7b) as well as the OEt content (Figure 7c). These can be explained by the side reactions in the chlorination process: the reaction between MGE and TiCl_4_ generated TiOEt_x_Cl_y_ species as a side product, and likewise, a high OEt content implied a high residue of TiOEt_x_Cl_y_ species that could not be removed by repetitive washing. Simultaneously, the occurrence of transesterification between DBP and TiOEt_x_Cl_y_ species led to the formation of DEP, thus providing a rationalization for the observed inverse correlation. Regarding the physical features, a larger mesopore volume contributed to enlarging the total pore volume (Figure 7d), and catalysts with a large particle size tended to have a small pore volume (Figure 7e). As for the attribution of physical features to the chemical composition, it was evidenced that the increase in particle size (Figure 7f) and micropore volume (Figure 7g) accompanied the increase in the OEt content, while the increase in the mesopore (Figure 7h) reduced it. One conceivable scenario might be that the removal of TiOEt_x_Cl_y_ species is regulated by the diffusion process: the TiOEt_x_Cl_y_ by-product, once formed, necessitates diffusion before its removal, wherein a substantial pore volume characterized by a relatively large size coupled with a small particle size, facilitates this diffusion mechanism, i.e., more efficient removal. From these correlations that stress the interdependence of catalyst features, it becomes clear that the alteration of a single parameter inevitably affects others, emphasizing the significance of a robust catalyst library to unveil the structure–performance relationship through comprehensive statistical analysis.

**Figure 8:**
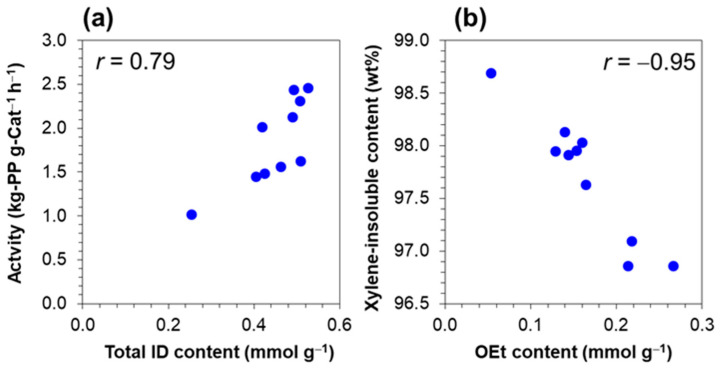
Relationships (**a**) between the total internal donor content and propylene polymerization activity, and (**b**) between the OEt content and xylene-insoluble content of resultant polymer.

Propylene polymerization was performed in a slurry phase to evaluate the performance of the catalysts. Table 4 summarizes the catalytic activity and xylene-insoluble content (XI) of the resultant polymer. Notably, all catalysts demonstrated an activity range akin to that of the MGE-based catalysts prepared using traditional glassware methods [33,34]. Previously, our studies indicated a tendency for polymerization activity to increase with the decrease in particle size [34,35]. However, this trend no longer holds with the expanded dataset, indicating that the correlation is dependent on selected samples. Conversely, the tendency for increasing the catalytic activity with a greater donor content (Figure 8a) was found to be consistent with previous findings, emphasizing the role of a donor in promoting polymerization. Furthermore, a distinct inverse correlation was observed between the OEt content and XI (Figure 8b). This agreed with our prior report for Ziegler-Natta catalysts prepared using different types of internal donors, in which the titanium alkoxy by-products were found to be the contributing factor to the increase in the amorphous fraction in polypropylene [36]. Correlations between the catalyst features and those between the catalyst features and performance, not only provide valuable insights but also stress the complexity and multifaceted nature of the catalytic system. The protocol established in this study holds promise as a foundation for expanding the catalyst library and facilitating more understanding through advanced statistical analysis methods.

## 4. Conclusions

In this work, we introduced a custom-designed reactor setup and synthetic protocol for the 12-parallel synthesis of magnesium ethoxide-based Ziegler-Natta catalysts. By adopting a miniature reaction vessel featuring a magnetic suspended stirrer, the synthetic scale was successfully reduced by over tenfold compared to typical laboratory-scale synthesis, leading to a significant reduction in the chemicals used and wasted. Given the multi-step nature of Ziegler-Natta catalyst synthesis, it is important to balance instrumental costs with the benefits of automation. Specifically, we strategically prioritize automation for critical synthetic steps, such as TiCl_4_ addition and temperature control, while maintaining manual operation for other aspects of the process. This approach made the system flexible for a broader range of conditions, as well as for other chemical synthesis applications at an affordable cost. With the developed protocol, we demonstrated that twelve catalysts synthesized in parallel reproduced each other well, ensuring consistency and reliability of the synthesis. Furthermore, the multifaceted nature of the catalyst and the complicated interplay in olefin polymerization emphasized the significance of a robust catalyst library. This is crucial for unveiling the structure–performance relationship through comprehensive statistical analysis, wherein the developed protocol shows promise as a foundation for catalyst library generation.

## Figures and Tables

**Table 1:** Particle characteristics and chemical composition of catalysts synthesized in parallel under identical conditions.

Run No.	Particle Characteristic ^a^					Chemical Composition				
	*D*_10_ (µm)	*D*_50_ (µm)	*D*_90_ (µm)	RSF	Circularity	Ti ^b^ (mmol g^−1^)	DBP ^c^ (mmol g^−1^)	DEP ^c^ (mmol g^−1^)	Total ID ^c^ (mmol g^−1^)	OEt ^c^ (mmol g^−1^)
P1	38.4	44.0	50.9	0.28	0.76	0.48	0.40	0.10	0.51	0.20
P2	38.1	43.4	49.2	0.26	0.71	0.48	0.43	0.09	0.52	0.22
P3	39.8	44.8	50.3	0.23	0.7	0.44	0.41	0.11	0.52	0.18
P4	38.5	43.6	48.4	0.23	0.76	0.46	0.46	0.09	0.55	0.22
P5	37.8	42.7	48.2	0.24	0.71	0.42	0.45	0.09	0.54	0.20
P6	38.6	44.5	51.0	0.28	0.74	0.44	0.43	0.09	0.51	0.24
P7	38.6	45.0	50.4	0.26	0.72	0.44	0.45	0.09	0.55	0.22
P8	37.9	42.9	48.7	0.25	0.68	0.46	0.42	0.11	0.53	0.18
P9	37.8	43.2	49.0	0.26	0.83	0.48	0.41	0.11	0.52	0.22
P10	36.9	42.6	49.1	0.29	0.76	0.46	0.40	0.10	0.50	0.20
P11	36.3	42.6	48.4	0.28	0.74	0.44	0.38	0.09	0.47	0.20
P12	38.4	44.0	50.9	0.28	0.76	0.48	0.41	0.09	0.50	0.16
AVG ^d^	38.1	43.6	49.5	0.26	0.74	0.46	0.42	0.10	0.52	0.20
STD ^d^	0.89	0.85	1.08	0.02	0.04	0.020	0.023	0.008	0.021	0.025
STE ^d^	0.26	0.25	0.31	0.01	0.01	0.006	0.007	0.002	0.006	0.007

Particle Characteristic ^a^ acquired from SEM images using ImageJ software. *D*_10_, *D*_50_ and *D*_90_ are the particle sizes at the accumulated number of 10%, 50% and 90%, respectively. RSF (relative span factor) and circularity are calculated using RSF = (*D*_90_ − *D*_10_)/*D*_50_, and circularity = (4π × area)/Perimeter^2^; ^b^ determined based on a colorimetric method using UV-vis; ^c^ analyzed using ^1^H NMR, in which DBP, DEP, Total ID and OEt are abbreviated from di-*n*-butyl phthalate, diethyl phthalate, total internal donor and ethoxy, respectively; ^d^ AVG, STD and STE stand for average, standard deviation and standard error, respectively.

**Table 2:** Particle characteristics and chemical composition of catalyst samples synthesized from different MGE sources.

Sample	MGE Source	Particle Characteristic					Catalyst Chemical Composition				
		*D*_10_ (µm)	*D*_50_ (µm)	*D*_90_ (µm)	RSF	Circularity	Ti (mmol g^−1^)	DBP (mmol g^−1^)	DEP (mmol g^−1^)	Total ID (mmol g^−1^)	OEt (mmol g^−1^)
CAT1	MGE-STD	39.2	45.3	54.6	0.34	0.84	0.62	0.46	0.07	0.52	0.14
CAT2	MGE-MND	40.7	48.0	59.9	0.40	0.81	0.58	0.44	0.05	0.49	0.15
CAT3	MGE-MGI	36.8	47.2	59.4	0.48	0.76	0.49	0.35	0.07	0.42	0.13
CAT4	MGE-DES	29.7	34.8	44.5	0.43	0.82	0.29	0.15	0.10	0.25	0.05
CAT5	MGE-MLA	37.0	43.0	52.9	0.37	0.81	0.59	0.44	0.07	0.51	0.16
CAT6	MGE-DCA	46.7	54.9	62.7	0.29	0.84	0.61	0.44	0.05	0.49	0.16
CAT7	MGE-PPA	47.2	58.6	67.3	0.34	0.84	0.72	0.39	0.07	0.46	0.22
CAT8	MGE-HXA	48.2	57.7	67.4	0.33	0.81	0.73	0.35	0.05	0.40	0.21
CAT9	MGE-ACA	72.5	87.7	102.4	0.34	0.77	0.74	0.47	0.03	0.51	0.27
CAT10	MGE-OLA	51.6	58.8	66.0	0.24	0.75	0.49	0.37	0.05	0.42	0.14
CAT8 *	MGE-HXA	51.3	59.3	68.7	0.29	0.74	0.73	0.38	0.05	0.43	0.24
CAT9 *	MGE-ACA	72.7	88.1	102.6	0.34	0.77	0.73	0.50	0.04	0.54	0.29

The * symbol indicates the reproduction test.

**Table 3:** Pore volume of catalyst samples acquired via N_2_ adsorption/desorption experiments.

Catalyst	Total Pore Volume ^a^ (cm^3^ g^−1^)	Micropore ^b^ (cm^3^ g^−1^)	Mesopore ^c^ (cm^3^ g^−1^)
CAT1	0.284	0.061	0.223
CAT2	0.302	0.071	0.230
CAT3	0.342	0.068	0.273
CAT4	0.354	0.033	0.322
CAT5	0.335	0.072	0.262
CAT6	0.283	0.066	0.217
CAT7	0.251	0.080	0.171
CAT8	0.230	0.071	0.159
CAT9	0.192	0.067	0.125
CAT10	0.233	0.045	0.188

Total Pore Volume ^a^ analyzed from the adsorption branch using the cumulative pore volume based on the NL-DFT method; ^b^ cumulative volume of pores with the pore width smaller than 2 nm; ^c^ cumulative volume of pores with the pore width larger than 2 nm.

**Table 4:** Propylene polymerization performance and xylene-insoluble content of resultant polymer samples.

Catalyst Sample	Activity ^a^ (kg-PP g-Cat^−1^ h^−1^)	XI (wt%)
CAT1	2.46	98.13
CAT2	2.44	97.96
CAT3	2.01	97.95
CAT4	0.98	98.70
CAT5	2.31	98.03
CAT6	2.13	97.63
CAT7	1.56	97.10
CAT8	1.45	96.86
CAT9	1.63	96.86
CAT10	1.49	97.92

Activity ^a^ polymerization condition: heptane = 300 mL; TEA = 3 mmol; C-donor = 0.3 mmol; P = 0.4 MPa; T = 50 °C; t = 1 h; catalyst amount = 15–20 mg.

## Data Availability

The data presented in this study are available on request.
